# Differential Diagnosis—Chicken Pox—Smallpox

**Published:** 1918-05

**Authors:** Edward Clark

**Affiliations:** Buffalo, N. Y.; Sanitary Supervisor New York State Dept. Health


### Differential Diagnosis—Chicken Pox—Smallpox.

By EDWARD CLARK, M. D., Buffalo,

Sanitary Supervisor New York State Dept. Health.

The eruption in smallpox is a deep-seated eruption involving the cutis vera, while the eruption in chicken pox involves the epidermis only or at best the superificial layers of the cutis vera.

The eruption in smallpox presents certain well defined and typical stages, and the lesions in a certain area are always of one distinct type, that is, they are always either papules, vesicles or pustules. On the other hand, in chicken pox the lesions in a certain small area, even when in close proximity to each other, are of various types. In an area no larger than half a dollar, in chicken pox, we may find macules, papules, vesicles, pustulated and dessicated vesicles in close proximity. The eruption in chicken pox comes in successive and reappearing crops in the same area. This never occurs in smallpox. When the papules begin to show themselves in small pox all of the papules that will occur in a certain area show themselves in a very short time and do not successively reappear. The areola surrounding the vesicle or pustule of smallpox is indurated, elevated, hard to the touch and of a carmine red color. In chicken pox the areola surrounding the vesicle is not indurated except where the skin is drawn tight over a bony surface, as the forehead, which produces what some authors call a shot-like feel; this fact alone leads to many errors in diagnosis for the authors have inculcated the idea that this shot-like feel is found only in smallpox. It occurs in measles, acne, drug rashes, and even in rashes produced by gastro-intestinal disturbances in those locations where the skin is close to the bony tissues. The color of the areola in chicken pox is more of an orange red than a carmine, and when the vesicle breaks down black crusts occur instead of the yellow or gray scab which we see in smallpox. The crusts in smallpox are seldom very dark except in the hemorrhagic type or in the cases where scratching or irritation has produced bleeding. The shape of the vesicle or pustule in smallpox is generally almost perfectly cylindrical, and before umbilication takes place, it stands out prominently like a lentil or split pea on the skin. In chicken pox the vesicle may be irregular in shape or distinctly ovid, which type is frequently seen on the trunk of the body, especially on the abdomen and loins.

As to location, in smallpox the eruption appears first on the face, particularly the forehead and nose, then on the hands, then on the feet, lastly on the trunk. The disease shows a decided predilection for the face, hands and feet. Except in very severe cases, there is not much eruption on the trunk, and generally it is almost entirely absent in the axillary spaces. It occurs on the soles of the feet, the palms of the hands, and on both the hard and soft palate. On the soles of the feet and the palms of the hands before the papule shows on the surface it may be felt through the thick skin, giving to the sense of touch the feeling of a small foreign body imbedded therein. Chicken pox, especially in cases where the eruption is profuse, shows a predilection for the trunk of the body rather than for the face, hands and feet, and the eruption here may occur simultaneously with or before the eruption shows itself on the face or hands. This is especially true in cases of chicken pox in the adult. If you close your eyes, and with your finger or thumb, properly protected, break one of the vesicles in smallpox and run your finger back and forth over it, you will still feel the hard, indurated base of the lesion. If you do this in chicken pox, you will detect a decided absence of this hard, indurated base.

It would seem that many authors and physicians believe that chicken pox seldom, if ever, occurs in adults. This is absolutely false teaching, as verified by my experience. I have seen in the past five years over three hundred cases of chicken pox in adults. When it occurs in adults, the eruption is generally much more profuse than it is with children, and at times is found on the palms of the hands, on the soles of the feet and on the hard and soft palate, also on the scalp.

As a rule, when there is smallpox, chicken pox is generally very prevalent, and I have seen chicken pox and smallpox in different members of the same family at the same time, each presenting its own peculiar diagnostic characteristics.

If any one therapeutic measure has been proven by long years of experience of incalculable value to the human race, it is vaccination for the prevention of smallpox. The statistics of the world relating to smallpox and vaccination, indisputably prove that more than 90 per cent of all cases of smallpox occur in persons who have never been successfully vaccinated, and that less than 10 per cent of all cases have occurred in persons who have been successfully vaccinated at any time during their lives. In view of these well-established facts, it is an absolute waste of time and effort to enter into any discussion at this late day as to the value of proper vaccination as a prophylactic measure against smallpox. I am radically opposed to all spasmodic efforts in vaccination; by this I mean that it is unwise to let the people in any community go unvaccinated until an epidemic of smallpox makes its appearance. If this rule were followed, there would be some communities where in the course of a generation no vaccination whatever would be done, with the result that the entire community would be a distinct menace to the surrounding country and state, if smallpox should make its appearance. My firm belief is that all children, unless physically incapacitated, should be vaccinated in the first two years of their life, or at least before they are allowed to enter a public or private school.

In conclusion I want to emphasize the fact that much of the well founded opposition to vaccination has been brought about and fostered by carelessness and indifference on the part of the medical profession toward this important and beneficent protective, by the careless and slipshod method in which the operation is performed by many physicians who can not plead ignorance as a cause of either indifference or neglect. Vaccination is a surgical procedure, and should be performed and cared for just as aseptically as any other surgical operation.

---

**Fracture of Atlas Without Medullary Symptoms.** J. and A. Boeckel, Lyon Med., Oct., have collected 31 cases of cervical fractures, including 14 of the atlas and axis, observed and treated since the development of radiography, which have not resulted in a single death. On the other hand, they have collected 36 cases of cervical fracture, including three before the advent of radiography, of which total, 17 involved the atlas and axis, with a total mortality of 22 (64%). The present case was that of a soldier wounded in the neck at Mort Homme. A mastoid fracture was discovered and trephined, recovery occurring without any special symptom except diminution of hearing. A small metallic splinter was extracted from the wound. During his stay in the hospital, he occasionally had formication in the fingers, some brief cramps and discoloration of the fingers. The reflexes were normal. X-ray examination showed a large shell fragment a finger's breadth from the right mastoid and a fracture and outward displacement of the posterior portion of the atlas. After extraction, it was found that the dressing of the wound sufficed to hold the bone in place. Rotation and flexion of the head were somewhat painful but not markedly restricted. The authors have devoted a chapter of a book to cervical fractures by missiles in which are reported 15 cases, 10 radiographed, with a mortality of 40%, from special complications, mostly haemorrhage.

